# Development of measurable indicators to enhance public health evidence-informed policy-making

**DOI:** 10.1186/s12961-018-0323-z

**Published:** 2018-05-31

**Authors:** Valentina Tudisca, Adriana Valente, Tommaso Castellani, Timo Stahl, Petru Sandu, Diana Dulf, Hilde Spitters, Ien Van de Goor, Christina Radl-Karimi, Mohamed Ahmed Syed, Natasa Loncarevic, Cathrine Juel Lau, Susan Roelofs, Maja Bertram, Nancy Edwards, Arja R. Aro, on behalf of the REPOPA Consortium, Arja R. Aro, Maja Bertram, Christina Radl-Karimi, Natasa Loncarevic, Gabriel Gulis, Thomas Skovgaard, Mohamed Ahmed Syed, Leena Eklund Karlsson, Mette W. Jakobsen, Ien A. M. van de Goor, Hilde Spitters, Timo Ståhl, Riitta-Maija Hämäläinen, Razvan M. Chereches, Diana Dulf, Petru Sandu, Elena Bozdog, Adriana Valente, Tommaso Castellani, Valentina Tudisca, Fabrizio Bianchi, Liliana Cori, Nancy Edwards, Sarah Viehbeck, Susan Roelofs, Christopher Anderson, Torben Jørgensen, Charlotte Glümer, Cathrine Juel Lau

**Affiliations:** 1 The National Research Council of Italy (CNR), Rome, Italy; 2 The National Institute for Health and Welfare (THL), Tampere, Finland; 3 Babeș-Bolyai University (BBU), Cluj-Napoca, Romania; 4 Tranzo, Tilburg University, Tilburg, The Netherlands; 5 Unit for Health Promotion Research, University of Southern Denmark (SDU), Odense, Denmark; 6 Primary Health Care Corporation, Doha, Qatar; 7 Center for Clinical Research and Disease Prevention, previously called Research Centre for Prevention and Health (RCPH), Bispebjerg and Frederiksberg Hospital, The Capital Region, Copenhagen, Denmark; 8 Ottawa University (uOttawa), Ottawa, ON Canada

**Keywords:** Evidence-informed policy-making, indicators, physical activity, Delphi methodology, co-production of knowledge, public health

## Abstract

**Background:**

Ensuring health policies are informed by evidence still remains a challenge despite efforts devoted to this aim. Several tools and approaches aimed at fostering evidence-informed policy-making (EIPM) have been developed, yet there is a lack of availability of indicators specifically devoted to assess and support EIPM. The present study aims to overcome this by building a set of measurable indicators for EIPM intended to infer if and to what extent health-related policies are, or are expected to be, evidence-informed for the purposes of policy planning as well as formative and summative evaluations.

**Methods:**

The indicators for EIPM were developed and validated at international level by means of a two-round internet-based Delphi study conducted within the European project ‘REsearch into POlicy to enhance Physical Activity’ (REPOPA). A total of 82 researchers and policy-makers from the six European countries (Denmark, Finland, Italy, the Netherlands, Romania, the United Kingdom) involved in the project and international organisations were asked to evaluate the relevance and feasibility of an initial set of 23 indicators developed by REPOPA researchers on the basis of literature and knowledge gathered from the previous phases of the project, and to propose new indicators.

**Results:**

The first Delphi round led to the validation of 14 initial indicators and to the development of 8 additional indicators based on panellists’ suggestions; the second round led to the validation of a further 11 indicators, including 6 proposed by panellists, and to the rejection of 6 indicators. A total of 25 indicators were validated, covering EIPM issues related to human resources, documentation, participation and monitoring, and stressing different levels of knowledge exchange and involvement of researchers and other stakeholders in policy development and evaluation.

**Conclusion:**

The study overcame the lack of availability of indicators to assess if and to what extent policies are realised in an evidence-informed manner thanks to the active contribution of researchers and policy-makers. These indicators are intended to become a shared resource usable by policy-makers, researchers and other stakeholders, with a crucial impact on fostering the development of policies informed by evidence.

**Electronic supplementary material:**

The online version of this article (10.1186/s12961-018-0323-z) contains supplementary material, which is available to authorized users.

## Background

Despite nearly two decades of efforts to improve evidence-informed policy-making (EIPM) in public health, many gaps remain. These gaps have been attributed to organisational and strategic factors influencing decisional processes, competing demands for resources, and public pressure and lobbying [1–4]. Challenging disconnects between research and policy-making processes, such as incompatible timeframes and competing values and interests [5–10], have also been described. While a number of tools and approaches have been developed to facilitate EIPM in public health [11–21], we registered a lack of availability of specific indicators for EIPM. The present study aimed to overcome this challenge by building a set of measurable indicators for EIPM in the field of public health. These indicators are intended to infer if and to what extent health-related policies are, or are expected to be, evidence-informed for the purposes of policy planning as well as formative and summative evaluations.

Several previous studies prepared the ground for building these indicators by critically reflecting on facilitators and barriers to EIPM [22]; giving value to the ‘knowledge transaction model’ approach over the ‘knowledge transfer’ model while building sustainability indicators [23]; identifying indicators to assess the performance of partnerships between researchers and policy-makers [24]; and developing indicators prioritised by the global community to provide concise information on the health situation and trends, including responses at national and global levels [25].

The innovative contribution of the current study is the development and validation of a set of measurable indicators specifically devoted to assess and support EIPM in the field of public health, intended to be jointly used by governmental policy-makers and researchers, but also by other stakeholders involved in various stages of the policy-making cycle.

The study was conducted within a 5-year European project called REPOPA (REsearch into POlicy to enhance Physical Activity), involving six European countries – Denmark, Finland, Italy, Romania, the Netherlands and the United Kingdom. The overall aim of the REPOPA project was to improve the integration of scientific research evidence and expert know-how in real world policy-making processes, establishing structures and best practices for health promotion and disease prevention [26], especially in inter-sectoral government administrational policies directed at physical activity promotion.

## Methods

We conducted the study in two phases. First, we developed a set of candidate indicators, based on two main inputs, namely literature findings and previous REPOPA research results [26, 27]. We then used the Delphi methodology [28–32] to identify other potential indicators and to validate the indicators in an international perspective.

The Delphi approach was chosen for three main reasons. First, it is participatory, engaging both scientists and policy-makers and, because of this, allows the capture of visions and values of the community for which the indicators are developed, as recommended in literature [33], instigating a joint activity and process involving both scientists and policy-makers [23, 34]. Second, we sought consensus among participants as we thought this would provide a more credible outcome for an international and inter-sectoral audience. Consensus was built through the rounds of the Delphi, wherein the initial group of collective responses of participants was used as an input in the second round of the Delphi, generating results that were co-produced through the group. Third, the Delphi is an efficient means to involve a wide range of experts from many countries at distance, with their ‘indirect interaction’ being mediated by the researchers conducting the study.

The main methodological process followed includes the development of an initial set of indicators as well as the preparation and implementation of the Delphi study to refine and integrate the initial set. These steps are described in detail in the following paragraphs.

### Developing the initial set of REPOPA indicators for EIPM

We defined a measurable indicator as an observable trait that is an objective measure of some other phenomenon difficult to estimate directly. Our focus was on indicators that could be used to assess if and to what extent a certain health policy is informed by evidence; we intended evidence in a wide sense, including research evidence, experiential evidence, and knowledge from stakeholders and target groups.

The initial set of indicators for EIPM was developed based on literature describing existing frameworks of EIPM processes, influences on these processes and constructs that were pertinent to indicator selection, and on previous REPOPA findings [26, 27].

As for the first input, we focused on two types of published frameworks. The first type described knowledge production and translation in policy-making environments. These frameworks, which concerned science-policy relationships and the use of science in policy-making [6, 35–37], explicated EIPM processes and stages, key actors and influences, and strategies to foster research knowledge use. The second type of framework examined EIPM processes within service delivery organisations [38–44], or knowledge translation processes between academics and knowledge users. This second set of frameworks specifically described stages of knowledge use in policy-making [41, 45, 46] and organisational factors that influenced users’ acquisition of research [24]. We also examined literature highlighting facilitators of EIPM [21, 22, 47–52] to select candidate indicators of these enablers. The European Responsible Research and Innovation framework [53] led us to include equity and inclusiveness elements in the indicators. Moreover, literature specifically focused on indicator development and/or validation in health and other policy sectors provided insights on the principles, criteria and processes to be considered while developing indicators [23–25, 54–58].

Our second input consisted of results from previous REPOPA research steps [26, 50, 58–66]. These findings informed the identification, selection and framing of some indicators. In particular, results highlighted the need for indicators that (1) were pertinent to a wide range of stakeholders working in different sectors and at different levels of government; (2) reflected how policy-makers mobilise internal and external networks to inform decisions about physical activity policies; and (3) took into account considerations about the diversity of target groups (including vulnerable populations). The process of building measurable indicators also involved converting tacit knowledge^1^ of the researchers of REPOPA Consortium into explicit knowledge, namely an ‘externalisation’ [67] that can be considered as a further input to the initial set of indicators for EIPM. This objective was achieved by means of both online and face-to-face meetings. In particular, the researchers were provided with a specific template for translating their findings into measurable indicators. To define the template structure, we considered previously reported findings [57]. After the first formulation, the proposed indicators were translated in terms of measurable indicators to infer the presence and the extent of EIPM in an objective way, applying the dimensions of ‘SMART’ indicators – specific, measurable, achievable/applicable, relevant, time-bound [33, 55].

Following the steps described above, we developed an initial set of 23 measurable indicators for EIPM to be used as the starting point for the two-round internet-based Delphi study.

### Preparing the two internet-based Delphi rounds

To prepare the two internet-based Delphi rounds, the initial set of indicators was organised in thematic domains and criteria were defined both for the type and number of panellists to be involved and the evaluation of the indicators.

### Defining thematic domains for the initial set of indicators

The 23 indicators were grouped into four thematic domains related to specific key aspects of EIPM [14, 24, 33, 40, 68–70]. These domains were as follows:

1. Human resources – Competences and Networking, focused on the possible kinds/types of human resources involved in a policy process (policy-makers, researchers, stakeholders and generic staff) and the skills they are required to have to contribute to EIPM;
2. Documentation – Retrieval/Production, concentrated on the retrieval and production of documents including scientific evidence during a policy process;
3. Communication and Participation, concerning both initiatives to inform several target groups during a policy process and engagement and consultation methodologies to gather knowledge from them, implying a bidirectional communication;
4. Monitoring and Evaluation, focused on the possible actors (researchers, policy-makers and other stakeholders) to be involved in monitoring and evaluating the use of scientific evidence in policies and related procedures to be adopted to achieve this aim.

### Selecting panellists

We aimed to involve an international group of panellists from the fields of health, physical activity and across sectors, with the roles of researchers, policy-makers (both civil servants and politicians) and other relevant stakeholders (e.g. non-governmental organisations). To ensure that different policy-making contexts in Europe were represented and to reach a wide perspective in the Delphi, we planned to have 12 panellists from each of the six REPOPA countries (termed ‘national panels’) and 10 additional panellists working at international level, for a total number of 82 panellists.

While composing each national panel, we aimed to get a balanced distribution of participants in terms of profession (researchers and policy-makers^2^), sectors (mainly public health, health policy, physical activity and sports, also with reference to disciplines like epidemiology, health economics, political science and social science) levels of policy-making (local, regional and/or national administrative levels), and gender.

We also aimed to include, in each national panel, at least one researcher with experience in science policy and at least one politician among the policy-makers.

Each country team of REPOPA researchers identified and informally contacted more than the scheduled 12 experts, starting from those satisfying the required criteria. We used a snowball sampling approach, asking these experts to suggest the names of other experts suitable for the research. A full list of potential participants was then generated for each country. Subsequently, each country team ranked the contacted experts according to their areas of competence and gradually built the planned panel, reaching the final number of 12 per country.

The panellists working in the international context were chosen among researchers and policy-makers from international organisations related to physical activity, public health, health promotion and policy innovation, e.g. WHO, the European Public Health Association, the Joint Research Centre, the AGE Platform Europe, the European Public Health Association.

The whole panel was thus assembled to include 82 panellists who agreed to take part in the Delphi study (for details see Additional file 1).

### Establishing evaluation criteria to rate indicators

We asked Delphi participants to assess the relevance and feasibility of the indicators. Relevance was defined as to the extent to which an indicator inferred the use of EIPM; feasibility was defined as the extent to which an indicator was applicable in EIPM assessment processes. Panellists scored each indicator using a four-point Likert scale (4 – very relevant, 3 - relevant, 2 – slightly relevant, 1 – not relevant; 4 – definitely feasible, 3 – probably feasible, 2 – slightly feasible, 1 – definitely not feasible).

**Fig. 1:**
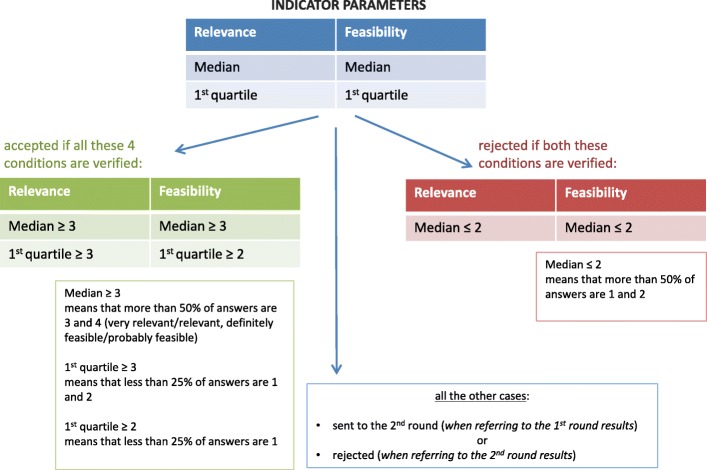
Algorithm for the selection of indicators based on the results of the two Delphi round

The algorithm developed for ‘accepting’ and ‘rejecting’ indicators, described in Fig. 1, was based on the calculation of medians and first quartiles of both relevance and feasibility.

To be included in the final set of indicators for EIPM, an indicator had to gather consensus on both high relevance and feasibility. Figure 1 shows the cut-off points for consensus we set for an indicator to be accepted or rejected on the left and right side, respectively. These conditions were valid for both the Delphi rounds, so that the indicators satisfying them already in the first round were either directly accepted or rejected and not listed in the second round. The central part of Fig. 1 shows the intermediate cases. Indicators that fell under this condition as a result of the first round were sent to the second round to be reconsidered. Indicators that fell under this condition as a result of the second round were finally rejected.

The indicators that were accepted, in either round, comprise the international set of REPOPA indicators for EIPM.

### Implementing the two internet-based Delphi rounds

#### The two Delphi questionnaires

The first- and second-round questionnaires were sent to the panellists in January and May 2015, respectively. Before the distribution, REPOPA researchers from each country team translated them to their national language from the agreed English master version; the panellists could answer either in English or in their native language. Moreover, the questionnaires were pilot-tested (in national language) in each country by two colleagues external to REPOPA project, checking the comprehensibility of the text of the questionnaire and the indicators (which form the bulk of the questionnaire), possible problems in interpreting questions, time to complete the questionnaire, possible problems with the online tool, and further comments.

The REPOPA Italian team, coordinating the Delphi study, defined a strategy of central and local management of the Delphi activities, and designed and arranged the web platform on *Limesurvey* to implement the Delphi process. The researchers of each country team managed the administration of the questionnaires in their own country supported by the Italian team and focused on keeping the country panellists on board by means of e-mail reminders or phone calls.

- *Questionnaire 1 description*

The first-round Delphi questionnaire presented the initial set of 23 indicators, organised in the four thematic domains previously described, and included an introduction on the aim of the indicators and a glossary for terms such as ‘EIPM’, ‘stakeholders’ or ‘vulnerable groups’, to help panellists to clearly understand the content of the indicators proposed.^3^

Panellists were asked to rate the relevance and feasibility of the indicators proposed and were invited to justify or elaborate their relevance and feasibility ratings with comments. In this questionnaire, panellists were also asked to suggest additional indicators to be included in the thematic domains.

- *Questionnaire 2 description*

The second-round Delphi questionnaire listed indicators along with histograms showing the frequencies for relevance and feasibility ratings obtained in the first round and summaries of comments (Additional file 2); this allowed panellists to take into account the first-round evaluations when they rescored the indicators.

A separate section of the second-round questionnaire included the new indicators suggested by the panellists in the first Delphi round. Additionally, for all indicators in the second round, panellists were invited to justify or elaborate their relevance and feasibility ratings with comments.

## Results

### Delphi panellists’ involvement

A total of 82 panellists, as planned, initially agreed to participate in the study, including 12 panellists per country (6 researchers and 6 policy-makers each from Denmark, Finland, Italy, Romania, the Netherlands and the United Kingdom) plus 10 international panellists, including 4 researchers and 6 policy-makers.

A total of 76 (92.7%) panellists answered the first round and 72 (87.8%) answered the second round, always keeping a balanced distribution between researchers and policy-makers.

### Developing the final set of REPOPA indicators for EIPM

- *Results of the first Delphi round*

Following the first round and using the initial set of 23 indicators proposed by the REPOPA team (Additional file 3), 14 indicators were accepted, 9 were sent to the second round for re-consideration and no indicators were discarded, according to the algorithm in Fig. 1 and based on panellists’ ratings.

The suggestions provided by panellists led to the development of 8 new indicators for EIPM to be rated by the panellists in round two (Additional file 4 lists the panellists’ comments that led to new indicators).

- *Results of the second Delphi round*

**Fig. 2:**
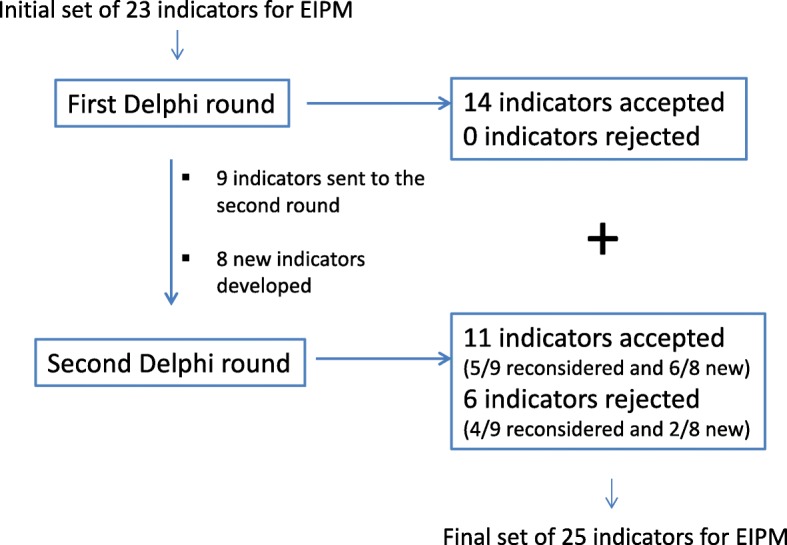
Schematic summary of the process of developing REPOPA indicators for evidence-informed policy-making (EIPM)

The second round led to the acceptance of another 11 indicators (in addition to the 14 previously accepted in the first round), including 5 indicators out of 9 from the initial set (that were neither accepted nor rejected in round 1), plus 6 new indicators out of the 8 proposed on the basis of suggestions given by the panellists in the first Delphi round. These 11 indicators were added to the 14 indicators already accepted in the first round to compose the final set of 25 REPOPA international indicators for EIPM (Fig. 2 and Additional file 5).

Table 1 shows the final set of indicators for evidence-informed policy-making (EIPM) organised in the four thematic domains.

**Table 1:** The final set of international REPOPA indicators for EIPM as a result of the two Delphi rounds, including both indicators from the initial set and new indicators proposed by panellists. The first and the second column include, respectively, the four thematic domains and the indicators, while the last column specifies at which round each indicator was accepted

Thematic domain	International REPOPA indicators for EIPM	Acceptance round
HUMAN RESOURCES	1. Staff with research experience working on the policy	1^st^ round
	2. Stakeholders working on the policy	2^nd^ round
	3. Partnerships with research institutions during the policy	1^st^ round
	4. Training courses on research issues and on EIPM for the staff working on the policy	1^st^ round
	5. Researchers with policy-making experience involved in the policy	2^nd^ round^a^
DOCUMENTATION	6. Procedures for ensuring a review of scientific literature relevant to the policy	1^st^ round
	7. Published scientific articles based on policy results	2^nd^ round
	8. Citation of peer-reviewed research articles in policy documents	2^nd^ round
	9. Citation of reports and other documents containing evidence in policy documents	1^st^ round
	10. Available evidence briefs for policy	2^nd^ round^a^
	11. Available reports on policy results from policy-making organisations of different municipalities/regions/countries	2^nd^ round^a^
COMMUNICATION AND PARTICIPATION	12. Initiatives to inform stakeholders during the policy	1^st^ round
	13. Initiatives to inform researchers during the policy	2^nd^ round
	14. Communication methods tailored for vulnerable groups likely to be impacted by the policy	2^nd^ round
	15. Engagement and consultation methodologies to gather knowledge from stakeholders during the policy	1^st^ round
	16. Engagement and consultation methodologies to gather knowledge from researchers during the policy	1^st^ round
	17. Engagement and consultation methodologies to gather knowledge from vulnerable groups during the policy	1^st^ round
	18. Budget for engagement and consultation methodologies	1^st^ round
	19. Communication competences among the staff who interacts with stakeholders	2^nd^ round^a^
	20. Initiatives for fostering knowledge sharing between different stakeholders	2^nd^ round^a^
	21. Initiatives for consulting target groups to get their perspectives	2^nd^ round^a^
MONITORING AND EVALUATION	22. Inclusion of EIPM in the evaluation criteria of the policy	1^st^ round
	23. Procedure for monitoring/evaluating the use of research evidence in the policy	1^st^ round
	24. Procedure for monitoring/evaluating the use of knowledge from stakeholders and target groups in the policy	1^st^ round
	25. Researchers working on the policy evaluation	1^st^ round

^a^Indicators developed based on first round panellists’ comments and evaluated in the second round

On the other hand, six indicators were deemed neither relevant nor feasible to be included in the final set of indicators, consisting of 4 indicators from the initial set and 2 new indicators proposed by panellists in the first Delphi round (Additional file 6). Most of these indicators (5 out of 6 indicators; indicators b–f in Additional file 6) were rejected on the basis of relevance, while only one (indicator a: Internships/fellowships provided by research institutions during the policy, Additional file 6) was rejected on the basis of both relevance and feasibility.

Table 1 and Additional file 6 show that all the indicators from the initial set of 23 indicators for EIPM related to acquiring^4^, citing^5^ and producing^6^ evidence in terms of documentation were included in the final set, and two more indicators^7^ attaining to the documentation thematic domain were proposed by panellists to specify the role of evidence briefs and reports on policy results from policy-making organisations at different territorial levels as relevant sources of knowledge.

All the indicators from the initial set related to the involvement of researchers in EIPM – from one-way and bidirectional exchange of knowledge with policy-makers^8^ to a more active role in the development of the policy and in the policy evaluation^9^ – were included in the final set. Moreover, the need for active involvement of policy-competent researchers was stressed by panellists with the proposal of a new indicator (Table 1, indicator 5: researchers with policy-making experience involved in the policy); this is complementary to the indicator related to the involvement of ‘staff with research experience’. On the other hand, indicator a ‘Internships/fellowships provided by research institutions during the policy’ (Additional file 6) was not considered relevant and feasible enough to be accepted. From panellists’ comments, it can be argued that the reason for discarding this indicator – which would be in line with WHO Regional Office for Europe’s recommendations [71] – might be their limited time duration, which does not meet the need of continuity in the relationship between researchers and policy-makers to foster EIPM.

The indicators implying a bidirectional knowledge exchange with stakeholders^10^ and their contribution to the policy (Table 1, indicator 2. Stakeholders working on the policy) were included in the final set and panellists further stressed the importance of communication with stakeholders by proposing three new indicators related to consulting target groups to get their perspective, to acquiring communication competences to interact with stakeholders and to fostering knowledge sharing also among different groups of stakeholders^11^. On the other hand, indicator e ‘Stakeholders working on the policy evaluation’ (Additional file 6) was not accepted by panellists, differently from the equivalent indicator referring to researchers (Indicator 25. Researchers working on the policy evaluation). In this case, what can be argued looking at panellists’ comments is that the prudence in attributing to stakeholders an evaluation role in policy can be linked to the risk of conflict of interests, together with the problem of establishing criteria to select the stakeholders to be involved.

The other remaining indicators discarded from the final set^12^ in Additional file 6 concern aspects that can be considered ‘procedural’ rather than ‘substantial’; most of them were related to budget issues. Based on panellists’ comments, it seems that some indicators were deemed not feasible due to the lack of dedicated budgets for EIPM.

## Discussion

The aim of this study was to develop a set of measurable indicators to infer the presence and the extent of EIPM in public health policies in order to fill a recognised gap. The study led to the development of 25 validated indicators for EIPM. Several features of these indicators are noteworthy. The international REPOPA indicators have been co-produced and validated by a panel working at international level, bringing together a large number of key experts geographically dispersed in six European countries, including also international organisations – a particularly relevant aspect if we consider that initiatives related to EIPM in the European Region are usually scattered and often stand-alone [71]. Moreover, the indicators were considered feasible and relevant for those working in an array of government sectors.

### Using the indicators to foster EIPM

The validated indicators for EIPM are intended to be used by decision-makers, researchers and other stakeholders at various stages of a policy-making process. Measurable indicators, by giving objective data, could help inform the design, implementation, and monitoring and evaluation of interventions to foster EIPM.

The indicators are particularly useful for evaluating public health and physical activity policies, either by the organisation responsible for the policy or by other stakeholders such as external evaluators or research institutes. They can support EIPM already during the agenda-setting phase, helping to identify crucial elements to infer the presence and the extent of EIPM to be considered. During the development of a policy, the indicators can be used to monitor enablers of or barriers to EIPM in the policy process, giving the measure of their occurrence, making it possible not only to assess whether, and to what degree, a policy is or is not being informed by evidence, but also to discover why and how, possibly allowing adjustments. The indicators can also be used to evaluate the extent of EIPM of an already implemented policy by the organisation responsible for the policy or other administrative or research bodies. Moreover, policy evaluations using the indicators can also provide valuable insights for future policy processes, also helping to infer if the policy has created new evidence.

Besides evaluation purposes, the indicators can form the basis for EIPM recommendations, implying actions that, if accomplished, would foster EIPM. The indicators may also be the basis for an intervention and for active, critical reflection on how and why EIPM might be addressed, as already shown in literature for other validated knowledge translation tools [14]. Therefore, the use of international REPOPA indicators for EIPM may support EIPM processes, ensuring not only that the policy is informed by evidence, but also that evidence is used instrumentally to support the selection of activities to be implemented [36, 65, 72, 73], and not selectively to justify an already made decision [1, 74].

The availability and use of the indicators proposed in this study may contribute to an organisational culture where extended value is given to the use of evidence for decisions. Others have shown that awareness of an indicator may lead policy-makers to perceive that a problem exists, to change the way they view the problem or to potentially focus the options they see as suitable solutions [75]. In this way, the indicators could also impact on stakeholders’ frameworks of thinking [56], and generate new norms for EIPM within governmentally broad social norms [54].

Furthermore, international REPOPA indicators are a valuable resource for EIPM beyond physical activity and the health field as they attain to transversal approaches to policy-making, enhancing their use for EIPM in other sectors. This is firstly due to the circumstance that all sectors use policy-making cycles with common elements. Moreover, this potential transferability of the indicators was enhanced by the variety of areas of competence and roles among Delphi panellists and the cross-sector approach that was followed and examined during the REPOPA project [26, 50, 63, 76].

### Implications for the uptake of REPOPA indicators

A first step towards the practical application of the international REPOPA indicators for EIPM has already been performed by testing them within national conferences held in the six REPOPA countries (to be presented in a later manuscript); based on these national conferences, evidence briefs and guidance resources for the use of the international REPOPA indicators were developed.

According to WHO Regional Office for Europe [71], many tools to support EIPM are already available but are not widely used, and more research and development should continue, including evaluation of new and existing tools [77]. Therefore, institutional support and incentives [78, 79] such as funding or other stimuli for the individuals to foster EIPM could be considered [80]. Health systems that provide strong incentives for dialogues between policy-makers and researchers through formalised processes and enabling structures and environments are actively facilitating knowledge generation. Formalised processes should include explicit incentives to demand and use evidence, as well as time and space for inter-linkages between policy-makers and researchers [43].

Specifically, we think that new approaches for institutionalisation of the indicators would be required, including what employees are rewarded for. A proposal would be to build in a requirement for an assessment of indicators on EIPM into routine job performance. Our suggestion related to the international REPOPA indicators, validated by this study, is to foster their joint use by policy-makers and researchers, as a way to encourage joint researcher–policy-maker teams – a possibility given by the fact that the indicators were jointly developed with the contribution of both researchers and policy-makers, also in line with the WHO recommendations of involving both researchers and policy-makers while developing tools [71]. Indeed, strengthening the interactions between researchers and policy-makers has been described as a potential solution to foster EIPM [22, 24, 81], to such an extent that, according to the WHO Regional Office for Europe, it should be required among the actions to foster EIPM for policy development by the establishment of a legal framework to support the use of evidence [71]. This issue is also reflected in several indicators retained in the final set of international REPOPA indicators that imply a relationship between researchers and policy-makers, also addressing the well-characterised communication gap between them [5, 14, 22, 24, 48, 69, 82]. Current views, which are reflected in the final set of indicators, suggest that EIPM-oriented communication between research and policy-makers should be systematic and continuous, consisting of a collaborative approach towards using knowledge in real-world settings, adapting research questions to policy needs and helping policy-makers to interpret research findings [6, 42, 43, 61, 72, 74, 78, 83–85].

Moreover, the future use of indicators is facilitated by the availability of a reliable version of the indicators in six country languages (in addition to English, Danish, Dutch, Finnish, Italian and Romanian). Although we did not provide back translation from the six national languages to English, the methodology adopted, involving two researchers external to REPOPA project per country for feedback regarding the comprehension and intelligibility of the questionnaires and indicators, can be considered as an initial step toward validation of the six versions of the set of indicators. This process of validation continued within the national conferences held in the six REPOPA countries and with the analysis and comparison of their results.

According to WHO Regional Office for Europe [71], existing evidence and tools for EIPM should be available in local languages and sharing lessons and learning from country experiences is important as an action to build EIPM capacities, in particular in assessing and comparing EIPM practices across countries.

Finally, according to the literature [86, 87], processes of interaction, discussion and exchange are more effective to promote learning than those based on summarising research, disseminating papers and commissioning reports. In this sense, as the REPOPA international Delphi process has the added value of being a research work and a first dissemination action at the same time, the REPOPA indicators have already started spreading.

### Strengths and limitations

Two main strengths of the study are the quality of the panel, including experts coming from different areas of competence and different geographical contexts, and the unusually high response rate obtained in both the first and second round of the Delphi (92.7% and 87.8%, respectively) [32, 88, 89]. Reaching this goal was supported by a coordination strategy that involved local management of country panellists by leads in each of the participating countries and Delphi coordinators supporting the local managing process. A possible limitation is that, in order to make the indicators adaptable to various contexts, we did not define specific units of measurement (e.g. Boolean, numerical, percentage values) and baselines (e.g. specific values to be reached to assess the presence of EIPM) to be assigned to each indicator – these should be established by the users with reference to the context of a specific health organisation or policy in a given territory. At the same time, psychometric assessment of the indicators could be performed in order to deeply understand latent factors in the indicators in view of improving their implementation in various health and research organisations, as reported in the literature for other tools [90].

### Implications for future research

Although the process of contextualising the indicators in different countries has already started by means of the national conferences held in the six European countries within the REPOPA project, further adaptations might be needed to enlarge the environments where this set of indicators can be applied, especially with reference to the specific contexts of resource scarcity and high burdens of disease in low- and middle-income countries, where evidence uptake to support effective and efficient health systems interventions is crucial to reduce health inequities [43] and EIPM might face specific barriers to be considered. In low-resource settings, among the variety of specificities to be kept in mind while dealing with EIPM, a further issue may concern the interface between national policies and the policies of international agencies.

At the same time, the implementation of the indicators within a specific health policy or organisation is still to be tested. Future empirical studies should test the proposed indicators in actual policy processes to further assess their usability and help to understand how to integrate them in the regular business of an organisation. This testing should also involve policies not strictly related to the health field in order to verify the transferability of the indicators to other sectors.

Finally, further implementation research would be required to examine processes necessary to stimulate the use of the indicators by researchers, policy-makers and other stakeholders.

## Conclusions

The study led to the development and validation at an international level of a set of measurable indicators specifically devoted to assess if and to what extent policies are realised in an evidence-informed manner. These indicators can also have a crucial impact on fostering the development of policies that are informed by evidence; they are intended to become a shared resource usable by policy-makers, researchers and other stakeholders determined to bringing evidence into policy development processes.

International REPOPA indicators embed several actions and aspects related to EIPM, including methodologies of communication with stakeholders, documentation issues, evaluation constraints and opportunities, and the possible creation of new evidence by policies. As a consequence, their use can support the establishment of routine processes to enhance EIPM, and foster innovation in key aspects of inter-sectoral policy-making.

## Additional files

Additional file 1: Description of the structure of the Delphi panel, including 82 panellists who agreed to take part in the study. (PDF 95 kb)

Additional file 2: Example of second round questionnaire to re-evaluate indicators that had not reached consensus on high relevance and feasibility in the first round. (PDF 35 kb)

Additional file 3: First Delphi round results for the initial set of 23 indicators developed by REPOPA researchers. The indicators highlighted in grey were sent to the second round for further evaluation. No indicators were rejected in the first round, according to the algorithm in Fig. 1. (DOCX 18 kb)

Additional file 4: Development of new indicators based on first Delphi round results. Summaries of the suggestions of new indicators by panellists (panellists’ country specified) in the left column and the corresponding formulation of measurable indicators in the right column. (DOCX 16 kb)

Additional file 5: Overview of the results of the two internet-based Delphi rounds in terms of medians and first quartiles of relevance and feasibility ratings for the indicators for EIPM developed in this study. (DOCX 21 kb)

Additional file 6: Indicators excluded from the final set. The first and second columns include, respectively, the four thematic domains and the indicators, while the third and fourth columns specify, respectively, at which round each indicator was rejected and the reason for rejection. (DOCX 15 kb)

## Abbreviations

EIPM: evidence-informed policy-making
EU: Europe
FIN: Finland
INT: International
IT: Italy
MAX: maximum
MIN: minimum
N/A: not applicable
NL: The Netherlands
REPOPA: REsearch into POlicy to enhance Physical Activity
RO: Romania
UK: The United Kingdom

## Footnotes

1 With ‘tacit knowledge’ we mean implicit and intuitive knowledge that is difficult to communicate, e.g. know-how acquired during practical experience, but we also include explicit knowledge that was not formalised in scientific papers or reports.

2 For policy-makers, we considered the following definition: “*people taking decisions about the proposal and/or implementation of a program, project or activity aimed to an institutional goal, and having responsibility on it*” [58, 91–93].

3 The first questionnaire comprised a further section concerning some multi-faceted aspects which influence EIPM, but too wide to be translated in terms of measurable indicators, which were presented under the label of ‘Towards complex indicators’ and were rated on their relevance. They will be the subject of a separate paper.

4 Indicator 6. Procedures for ensuring a review of scientific literature relevant to the policy.

5 Indicator 8. Citation of peer-reviewed research articles in policy documents; Indicator 9. Citation of reports and other documents containing evidence in policy documents.

6 Indicator 7. Published scientific articles based on policy results.

7 Indicator 10. Available evidence briefs for policy; Indicator 11. Available reports on policy results from policy-making organisations of different municipalities/regions/countries.

8 Indicator 4. Training courses on research issues and on EIPM for the staff working on the policy; Indicator 13. Initiatives to inform researchers during the policy; Indicator 16. Engagement and consultation methodologies to gather knowledge from researchers during the policy.

9 Indicator 1. Staff with research experience working on the policy; Indicator 25. Researchers working on the policy evaluation.

10 Indicator 12. Initiatives to inform stakeholders during the policy; Indicator 14. Communication methods tailored for vulnerable groups likely to be impacted by the policy; Indicator 15. Engagement and consultation methodologies to gather knowledge from stakeholders during the policy; Indicator 17. Engagement and consultation methodologies to gather knowledge from vulnerable groups during the policy.

11 Indicator 19. Communication competences among the staff who interacts with stakeholders; Indicator 20. Initiatives for fostering knowledge sharing between different stakeholders; Indicator 21. Initiatives for consulting target groups to get their perspectives.

12 Indicator b. Budget for scientific advice; Indicator c. Administrative procedures allowing timely employment of research staff and scientific advisors; Indicator d. Budget for producing/acquiring scientific publications; Indicator f. Budget for external evaluation of the policy in Additional file 6.
