# Supplementary material for: Development of measurable indicators to enhance public health evidence-informed policy-making
Source: Health Res Policy Syst. 2018 May 31;16:47. doi: 10.1186/s12961-018-0323-z (PMC5984390; doi:10.1186/s12961-018-0323-z)

Selected Delphi panel

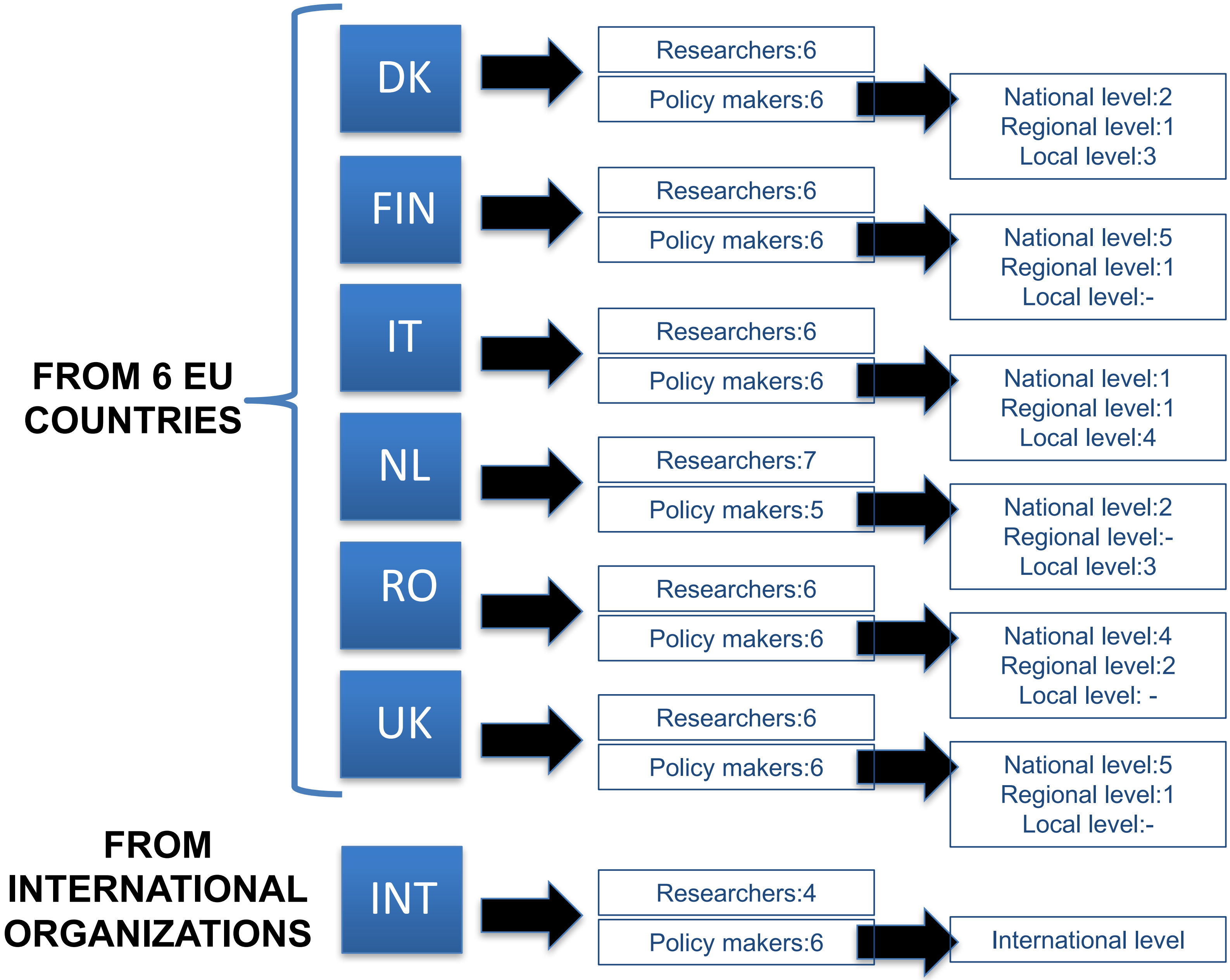

Supplement: Supplementary file 1 — Description of the structure of the Delphi panel, including 82 panellists who agreed to take part in the study. (PDF 95 kb) [file 12961_2018_323_MOESM1_ESM.pdf]
