# Supplementary material for: Development of measurable indicators to enhance public health evidence-informed policy-making
Source: Health Res Policy Syst. 2018 May 31;16:47. doi: 10.1186/s12961-018-0323-z (PMC5984390; doi:10.1186/s12961-018-0323-z)
Supplement: Supplementary file 2 — Example of second round questionnaire to re-evaluate indicators that had not reached consensus on high relevance and feasibility in the first round. (PDF 35 kb) [file 12961_2018_323_MOESM2_ESM.pdf]

1. Stakeholders working on the policy

1st round ratings (frequencies):

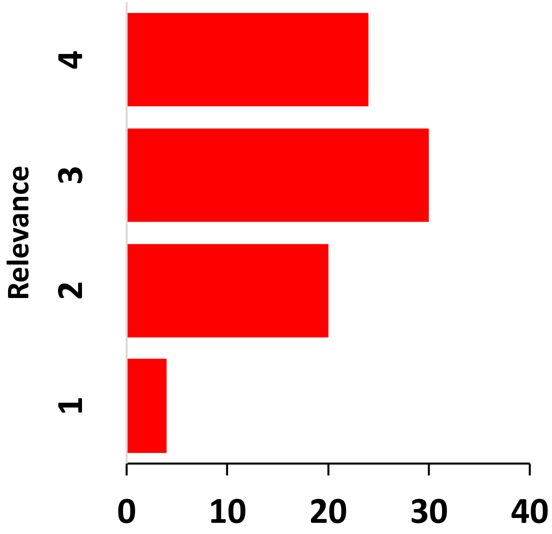

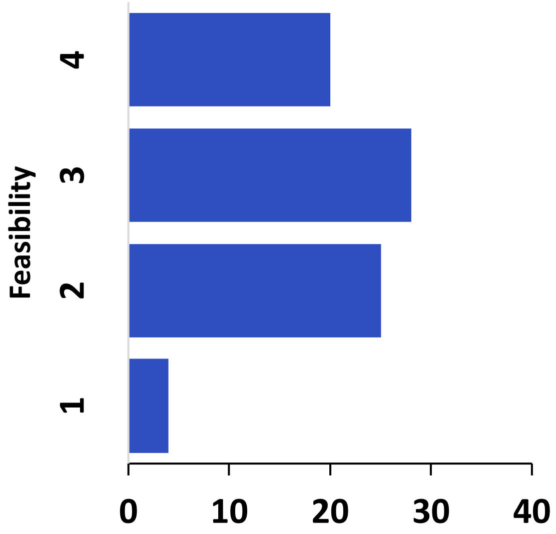

Summary of 1st round comments:

- stakeholders involvement in policy making is crucial in order to produce relevant policies and to implement them.
- stakeholders involvement may be problematic as they may not be qualified for their role and responsibility in the policy and as they may just push own interests.

Please rate again the relevance (how much the indicator is fit to infer the use of EIPM) and feasibility (how much the indicator is actually measurable). From 4 (max) to 1 (min). *
Please choose the appropriate response for each item:

	Relevance				Feasibility			
	4	3	2	1	4	3	2	1
**Stakeholders** working on the policy	◯	◯	◯	◯	◯	◯	◯	◯

GLOSSARY

Stakeholders: people and entities with interests in the policy, except the researchers, separately considered. E.g. enterprises, NGOs, associations (of patients, of citizens, of consumers...), other institutions (municipalities, health units...), practitioners (technicians, consultants...)
