# Supplementary material for: Development of measurable indicators to enhance public health evidence-informed policy-making
Source: Health Res Policy Syst. 2018 May 31;16:47. doi: 10.1186/s12961-018-0323-z (PMC5984390; doi:10.1186/s12961-018-0323-z)
Supplement: Supplementary file 3 — First Delphi round results for the initial set of 23 indicators developed by REPOPA researchers. The indicators highlighted in grey were sent to the second round for further evaluation. No indicators were rejected in the first round, according to the algorithm in Fig. 1. (DOCX 18 kb) [file 12961_2018_323_MOESM3_ESM.docx]

| **Thematic domain** | **INITIAL SET OF INDICATORS FOR EIPM** | **1^st^round results** |
| --- | --- | --- |
| **HUMAN RESOURCES** | Staff with research experience working on the policy | Accepted |
|  | Stakeholders working on the policy | Sent to the 2^nd^ round |
|  | Partnerships with research institutions during the policy | Accepted |
|  | Training courses on research issues and on EIPM for the staff working on the policy | Accepted |
|  | Internships/fellowships provided by research institutions during the policy | Sent to the 2^nd^ round |
|  | Budget for scientific advice | Sent to the 2^nd^ round |
| **DOCUMENTATION** | Procedures for ensuring a review of scientific literature relevant to the policy | Accepted |
|  | Published scientific articles based on policy results | Sent to the 2^nd^ round |
|  | Citation of peer-reviewed research articles in policy documents | Sent to the 2^nd^ round |
|  | Citation of reports and other documents containing evidence in policy documents | Accepted |
|  | Budget for producing/acquiring scientific publications | Sent to the 2^nd^ round |
| **COMMUNICATION AND PARTICIPATION** | Initiatives to inform stakeholders during the policy | Accepted |
|  | Initiatives to inform researchers during the policy | Sent to the 2^nd^ round |
|  | Communication methods tailored for vulnerable groups likely to be impacted by the policy | Sent to the 2^nd^ round |
|  | Engagement and consultation methodologies to gather knowledge from stakeholders during the policy | Accepted |
|  | Engagement and consultation methodologies to gather knowledge from researchers during the policy | Accepted |
|  | Engagement and consultation methodologies to gather knowledge from vulnerable groups during the policy | Accepted |
|  | Budget for engagement and consultation methodologies | Accepted |
| **MONITORING AND EVALUATION** | Inclusion of EIPM in the evaluation criteria of the policy | Accepted |
|  | Procedure for monitoring/evaluating the use of research evidence in the policy | Accepted |
|  | Procedure for monitoring/evaluating the use of knowledge from stakeholders and target groups in the policy | Accepted |
|  | Researchers working on the policy evaluation | Accepted |
|  | Stakeholders working on the policy evaluation | Sent to the 2^nd^ round |
