# Supplementary material for: Development of measurable indicators to enhance public health evidence-informed policy-making
Source: Health Res Policy Syst. 2018 May 31;16:47. doi: 10.1186/s12961-018-0323-z (PMC5984390; doi:10.1186/s12961-018-0323-z)
Supplement: Supplementary file 4 — Development of new indicators based on first Delphi round results. Summaries of the suggestions of new indicators by panellists (panellists’ country specified) in the left column and the corresponding formulation of measurable indicators in the right column. (DOCX 16 kb) [file 12961_2018_323_MOESM4_ESM.docx]

| **Suggestions or comments by panelists during round 1 that yielded new indicators** | **New indicator developed and introduced in round 2 of the Delphi*** |
| --- | --- |
| - Communication competences performing knowledge brokering (IT) - Experience of communicating policy and social marketing expertise (UK) - The interpretation and dissemination of policy is critical (UK) | Communication competences among the staff interacting with stakeholders |
| - The major obstacle is the lack of effective administrative procedures to hire personnel and manage resources (IT) | Administrative procedures allowing timely recruitment of research staff and scientific advisors |
| - Access to scientific evidence during the policy development process and availability of scientific based factsheets containing information relevant to the policy (NL) - Regular briefs evidence-based for policy and available evidence data (IT) - The procedures in EIPM include both the review of literature, but especially how to interpret the literature and formulation of recommendations based on it (DK) - Reviews/policy brief and meta analysis should be used more systematically (FIN) | Available evidence briefs for policy |
| - Courses about implementing and experiences from other institutions/municipalities/sectors (DK) - Issuing reports and documents for other administrations (IT) - The capacity of producing new evidence from previous policy results, the recognised responsibility of person/group able to interpret and apply research evidence seem to have priority in influencing EIPM (IT) - Importance should be given to experience and opinions of policy makers particularly with similar kinds of activities/programmes (UK) | Available reports on policy results from policy organizations of different municipalities/regions/countries |
| - Next to policy staff members with research experience, also researchers with policy experience (NL) | Researchers with policy making experience involved in the policy |
| - Collecting knowledge from the target group of the policy (NL) | Initiatives for consulting target groups to get their perspectives |
| - Having external evaluators is the optimal solution, because they are neutral (DK) | Budget for external evaluation of the policy |
| - Knowledge-sharing with stakeholders (DK) - The knowledge concerning the policy might be used and compared with the knowledge the stakeholders possess (NL) | Initiatives for fostering knowledge sharing between different stakeholders |

*developed by REPOPA researchers based on panelists’ comments
