# Supplementary material for: Development of measurable indicators to enhance public health evidence-informed policy-making
Source: Health Res Policy Syst. 2018 May 31;16:47. doi: 10.1186/s12961-018-0323-z (PMC5984390; doi:10.1186/s12961-018-0323-z)
Supplement: Supplementary file 5 — Overview of the results of the two internet-based Delphi rounds in terms of medians and first quartiles of relevance and feasibility ratings for the indicators for EIPM developed in this study. (DOCX 21 kb) [file 12961_2018_323_MOESM5_ESM.docx]

| **Indicators domain** | **Indicators** | **1^st^ round**  **Median**  **relevance** | **1^st^ round**  **Median**  **feasibility** | **1^st^ round**  **1st quartile**  **relevance** | **1^st^ round**  **1st quartile**  **feasibility** | **Status after the 1^st^ round** | **2^nd^ round**  **Median**  **relevance** | **2^nd^ round**  **Median**  **feasibility** | **2^nd^ round**  **1st quartile**  **relevance** | **2^nd^ round**  **1st quartile**  **feasibility** | **Status after the 2^nd^ round** |
| --- | --- | --- | --- | --- | --- | --- | --- | --- | --- | --- | --- |
| Human resources | Staff with research experience working on the policy | 3 | 3 | 3 | 2 | Accepted | n/a | n/a | n/a | n/a | Accepted |
|  | Stakeholders working on the policy | 3 | 3 | 2 | 2 | Sent to the 2^nd^ Round | 3 | 3 | 3 | 2 | Accepted |
|  | Partnerships with research institutions during the policy | 3 | 3 | 3 | 3 | Accepted | n/a | n/a | n/a | n/a | Accepted |
|  | Training courses on research issues and on EIPM for the staff working on the policy | 3 | 3 | 3 | 2 | Accepted | n/a | n/a | n/a | n/a | Accepted |
|  | Internships/fellowships provided by research institutions during the policy | 2,5 | 3 | 2 | 2 | Sent to the 2^nd^ Round | 2 | 2,5 | 2 | 2 | Rejected |
|  | Budget for scientific advice | 3 | 3 | 2 | 2 | Sent to the 2^nd^ Round | 3 | 3 | 2 | 2 | Rejected |
| Documentation | Procedures for ensuring a review of scientific literature relevant to the policy | 4 | 3 | 3 | 2 | Accepted | n/a | n/a | n/a | n/a | Accepted |
|  | Published scientific articles based on policy results | 3 | 3 | 2 | 2,75 | Sent to the 2^nd^ Round | 3 | 3 | 3 | 3 | Accepted |
|  | Citation of peer-reviewed research articles in policy documents | 3 | 3 | 2 | 3 | Sent to the 2^nd^ Round | 3 | 3 | 3 | 3 | Accepted |
|  | Citation of reports and other documents containing evidence in policy documents | 3 | 3 | 3 | 3 | Accepted | n/a | n/a | n/a | n/a | Accepted |
|  | Budget for producing/acquiring scientific publications | 3 | 3 | 2 | 2 | Sent to the 2^nd^ Round | 3 | 3 | 2 | 2 | Rejected |
| Communication & Participation | Initiatives to inform stakeholders during the policy | 3,5 | 3 | 3 | 2,75 | Accepted | n/a | n/a | n/a | n/a | Accepted |
|  | Initiatives to inform researchers during the policy | 3 | 3 | 2 | 2 | Sent to the 2^nd^ Round | 3 | 3 | 3 | 3 | Accepted |
|  | Communication methods tailored for vulnerable groups likely to be impacted by the policy | 4 | 2,5 | 2,75 | 2 | Sent to the 2^nd^ Round | 4 | 3 | 3 | 2 | Accepted |
|  | Engagement and consultation methodologies to gather knowledge from stakeholders during the policy | 4 | 3 | 3 | 2 | Accepted | n/a | n/a | n/a | n/a | Accepted |
|  | Engagement and consultation methodologies to gather knowledge from researchers during the policy | 3 | 3 | 3 | 2 | Accepted | n/a | n/a | n/a | n/a | Accepted |
|  | Engagement and consultation methodologies to gather knowledge from vulnerable groups during the policy | 3,5 | 3 | 3 | 2 | Accepted | n/a | n/a | n/a | n/a | Accepted |
|  | Budget for engagement and consultation methodologies | 3 | 3 | 3 | 2 | Accepted | n/a | n/a | n/a | n/a | Accepted |
| Monitoring & Evaluation | Inclusion of EIPM in the evaluation criteria of the policy | 4 | 3 | 3 | 2 | Accepted | n/a | n/a | n/a | n/a | Accepted |
|  | Procedure for monitoring/evaluating the use of research evidence in the policy | 3 | 3 | 3 | 2 | Accepted | n/a | n/a | n/a | n/a | Accepted |
|  | Procedure for monitoring/evaluating the use of knowledge from stakeholders and target groups in the policy | 3 | 3 | 3 | 2 | Accepted | n/a | n/a | n/a | n/a | Accepted |
|  | Researchers working on the policy evaluation | 3,5 | 3 | 3 | 3 | Accepted | n/a | n/a | n/a | n/a | Accepted |
|  | Stakeholders working on the policy evaluation | 3 | 3 | 2 | 2 | Sent to the 2^nd^ Round | 3 | 3 | 2 | 2 | Rejected |
| New indicators | Administrative procedures allowing timely employment of research staff and scientific advisors | n/a | n/a | n/a | n/a | n/a | 3 | 3 | 2 | 2 | Rejected |
|  | Researchers with policy making experience involved in the policy | n/a | n/a | n/a | n/a | n/a | 4 | 3 | 3 | 2 | Accepted |
|  | Available evidence briefs for policy | n/a | n/a | n/a | n/a | n/a | 4 | 3 | 3 | 3 | Accepted |
|  | Available reports on policy results from policy making organizations of different municipalities/regions/countries | n/a | n/a | n/a | n/a | n/a | 3 | 3 | 3 | 2,75 | Accepted |
|  | Communication competences among the staff who interacts with stakeholders | n/a | n/a | n/a | n/a | n/a | 4 | 3 | 3 | 2 | Accepted |
|  | Initiatives for fostering knowledge sharing between different stakeholders | n/a | n/a | n/a | n/a | n/a | 3 | 3 | 3 | 2 | Accepted |
|  | Initiatives for consulting target groups to get their perspectives | n/a | n/a | n/a | n/a | n/a | 4 | 3 | 3 | 2 | Accepted |
|  | Budget for external evaluation of the policy | n/a | n/a | n/a | n/a | n/a | 3 | 3 | 2 | 2 | Rejected |
