# Supplementary material for: Development of measurable indicators to enhance public health evidence-informed policy-making
Source: Health Res Policy Syst. 2018 May 31;16:47. doi: 10.1186/s12961-018-0323-z (PMC5984390; doi:10.1186/s12961-018-0323-z)
Supplement: Supplementary file 6 — Indicators excluded from the final set. The first and second columns include, respectively, the four thematic domains and the indicators, while the third and fourth columns specify, respectively, at which round each indicator was rejected and the reason for rejection. (DOCX 15 kb) [file 12961_2018_323_MOESM6_ESM.docx]

| **Thematic domain** | **INDICATORS EXCLUDED FROM THE FINAL SET** | **Rejection round** | **Reason for rejection** |
| --- | --- | --- | --- |
| **HUMAN RESOURCES** | a. Internships/fellowships provided by research institutions during the policy | 2^nd^ round | Relevance and feasibility |
|  | b. Budget for scientific advice | 2^nd^ round | Relevance |
|  | c. Administrative procedures allowing timely employment of research staff and scientific advisors | 2^nd^ round* | Relevance |
| **DOCUMENTATION** | d. Budget for producing/acquiring scientific publications | 2^nd^ round | Relevance |
| **COMMUNICATION AND PARTICIPATION** | n/a | n/a | n/a |
| **MONITORING AND EVALUATION** | e. Stakeholders working on the policy evaluation | 2^nd^ round | Relevance |
|  | f. Budget for external evaluation of the policy | 2^nd^ round* | Relevance |

*indicators developed based on first round panelists’ comments and evaluated in the second round
